# Undifferentiated Connective Tissue Disease in Pregnancy: A Topic Yet to be Explored

**DOI:** 10.3389/fphar.2022.820760

**Published:** 2022-01-20

**Authors:** Caterina Serena, Sara Clemenza, Serena Simeone, Sara Zullino, Serena Ottanelli, Marianna Pina Rambaldi, Silvia Vannuccini, Felice Petraglia, Federico Mecacci

**Affiliations:** ^1^ High Risk Pregnancy Unit, Careggi University Hospital, Florence, Italy; ^2^ Department of Biomedical, Experimental and Clinical Sciences, Careggi University Hospital, University of Florence, Florence, Italy

**Keywords:** undifferentiated connective tissue disease, pregnancy, antinuclear antibodies, flare, treatment, obstetric complications, autoimmune disease

## Abstract

Undifferentiated connective tissue disease (UCTD) is characterized by signs and symptoms suggestive of a connective tissue disease (CTD), but not fulfilling criteria for a specific CTD. Although UCTD is probably the most common rheumatic disease diagnosed in pregnant women, data about disease course during pregnancy and perinatal outcomes are very limited. Compared to other CTDs, UCTD seems to have milder clinical manifestations in pregnancy. Its natural history is related to disease activity at conception. In fact, if the disease is in a state of remission or minimal activity at conception, pregnancy outcomes are generally good. On the contrary, patients who become pregnant in a moment of high disease activity and/or who have multiple antibodies positivity show an increased risk of disease flares, evolution to a definite CTD and obstetric complications, such as fetal growth restriction, preeclampsia and preterm birth. Therefore, a preconception assessment is essential in women with UCTD to evaluate maternal and fetal risks, to initiate interventions to optimize disease activity, and to adjust medications to those that are least harmful to the fetus. The aim of the present study was to review the available literature about pregnancy course, maternal and fetal outcomes and therapeutic approaches of pregnant women with UCTD.

## Introduction

Autoimmune connective tissue diseases (CTDs) are more common in women than in men, especially during the childbearing age (Marder et al., 2016; Arese et al., 2019).

The relationship between autoimmune disease and reproduction is bidirectional: the disease can affect women’s reproductive health and pregnancy can affect the course of the disease (Cervera and Balasch, 2008). Historically, women with autoimmune disorders have been discouraged from having children, due to the possible risk of disease flare and adverse perinatal outcomes. However, the impact of pregnancy on the disease course and the impact of the disease on pregnancy course vary according to the type of autoimmune disorder (Piccinni et al., 2016). It is well known that women with CTDs have an increased risk of pregnancy complications, such as miscarriage, preeclampsia (PE), fetal growth restriction (FGR), and preterm birth (PTB) (Mecacci et al., 2007; Østensen et al., 2015). Nevertheless, when an adequate preconception counseling, a good disease control before conception and a proper medical care are provided, safe and uneventful pregnancies may be obtained (Piccinni et al., 2016).

The most common CTDs include rheumatoid arthritis (RA), systemic lupus erythematosus (SLE), antiphospholipid antibody syndrome (APS), systemic sclerosis (SSc), primary Sjogren’s syndrome (PSS), and inflammatory myositis (Marder et al., 2016). For each one, standardized classification criteria have been developed. However, if the clinical and serological features do not fulfill any of these diagnostic criteria, the diagnosis of undifferentiated connective tissue disease (UCTD) will be formulated (Marwa and Anjum, 2021). UCTD is, therefore, an umbrella for a wide variety of diseases characterized by laboratory findings of autoimmunity and by signs and symptoms reported in other CTDs (Antunes et al., 2019).

To date, whether pregnancy may influence the disease course and whether the disease may influence pregnancy course, is poorly studied and no specific recommendations for clinicians exist (Zucchi et al., 2020).

The aim of the present study was to review the available literature about pregnancy course, maternal and fetal outcomes and therapeutic approaches of pregnant women with UCTD.

## Definition and Epidemiology of UCTD

Diagnostic criteria for UCTD were proposed in 1999, although they are still debated (Mosca et al., 1999; Antunes et al., 2019). According to them, stable UCTD should be diagnosed when all of the following criteria are present: 1) signs and symptoms indicative of a CTD, but not meeting the required criteria to diagnose a specific CTD, 2) positive antinuclear antibodies (ANA) detected on two separate measurements and 3) a disease duration of at least 3 years (Mosca et al., 2006). However, patients with recent onset of symptoms, shorter follow up and unclassifiable clinical picture should be also considered as having UCTD. In this group of patients, the undifferentiated condition may represent the “early phase” of a CTD and its recognition has a critical importance in term of disease monitoring and choice of treatment (Mosca et al., 2014). Recognizing CTD, such as SLE, at an early stage is thus important to avoid irreversible target-organ damage from occurring (Sciascia et al., 2022).

Although it is considered one of the most common rheumatic disorders, the exact prevalence and incidence of UCTD are unknown (Bourn and James, 2015; Elfving et al., 2016). It was suggested that its annual incidence varies from 41 to 149 per 100,000 adults (Deane and El-Gabalawy, 2014). Up to 90% of UCTD cases occur in women, largely between 32 and 44 years old (Marwa and Anjum, 2021).

**FIGURE 1:**
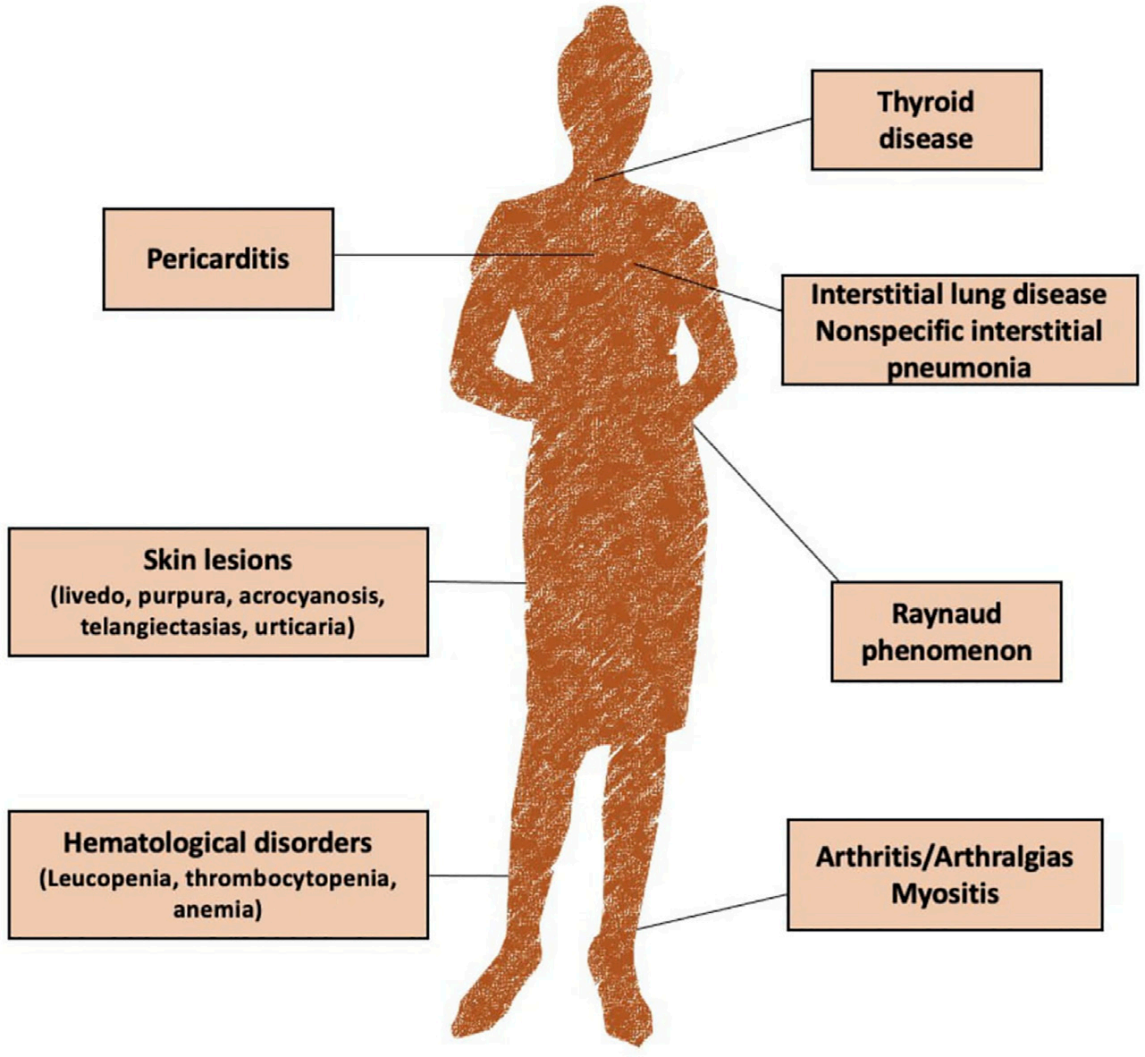
Main clinical features of undifferentiated connective tissue disease (UCTD).

Clinical presentation can vary widely among patients. The most common reported symptoms include arthralgia, skin lesions (livedo, purpura, acrocyanosis, telangiectasias, and urticaria), Raynaud phenomenon, mucocutaneous symptoms, arthritis, fever, non-specific interstitial pneumonia and thyroid disfunction (Mosca et al., 2012; Marwa and Anjum, 2021) (Figure 1).

It was estimated that about 30% of patients with UCTD will develop a specific CTD (Mosca et al., 2006; Vaz et al., 2009; Conti et al., 2010), while 21–87% will report a persistence in the undifferentiated state over time (Deane and El-Gabalawy, 2014; Pepmueller, 2016). Among the UCTD patients who evolved to CTDs, SLE is the most frequently reported diagnosis (Mosca et al., 2014).

The risk to develop a defined CTD seems to be higher during the first years after initial manifestations and in patient with multiple autoantibody positivity, such as anti-double-stranded DNA (dsDNA), antiphospholipid antibody (aPL), anti-Ro/SSA and anti-La/SSB (Mosca et al., 2002; Al Attia, 2006). Since evolution to a defined CTD can occur, it is necessary to follow up these patients regularly, especially during the first years from symptoms onset, or during conditions which may influence the course of an autoimmune disease, such as pregnancy (Mosca et al., 2006; Pepmueller, 2016). Interestingly, UCTD was reported to be the most frequently diagnosed systemic rheumatic disorder during the first trimester of pregnancy, with a prevalence of 2.5% (Spinillo et al., 2008a).

## How Pregnancy Can Affect the Disease Course?

During pregnancy, maternal immune system undergoes profound changes in order to accommodate the semiallogeneic fetus and to facilitate embryo implantation, as well as fetal growth, development and birth. However, these adaptive mechanisms do not compromise the maternal immune system function and do not make the mother and fetus more vulnerable to foreign insults (Yeung and Dendrou, 2019). A switch to a predominantly Th2-type cytokine profile plays a key role in the maintenance of immune tolerance toward allogeneic fetal antigens. Specific Th2, Th17/Th2 and Treg cells, produced during pregnancy, accumulate in the decidua and in maternal circulation and can regulate autoimmune responses, generally improving Th1/Th17-type autoimmune diseases (e.g., RA, multiple sclerosis, Grave’s disease and Hashimoto disease) and worsening Th2-type autoimmune disease (e.g., SLE, SSc) (Piccinni et al., 2016). Previous findings showed that a immunoregulatory imbalance between Th17 cells and Treg cells may drive the progression of UCTD to a definitive CTDs (Szodoray et al., 2013).

However, the impact of pregnancy on the immune system of patients with UCTD and on the disease course has been little investigated.

It was shown that pregnancy and puerperium may trigger disease flares and evolution to a definite CTDs (Castellino et al., 2011; Yang et al., 2020). Severe disease activity was reported in about 2% of pregnant women with UCTD, which typically show a more benign course compared to other CTDs, such as SLE (Kaufman et al., 2021).

In a case-control study, (Spinillo et al., 2008b) found that during pregnancy UCTD progresses to a definite CTD in 4.9% of cases. The most frequently reported symptoms included photosensitivity (70.7%), erythema and/or malar rash (43.9%), and Raynaud’s phenomenon (46.3%). Moreover, 24.4% of them experienced a disease flare that required treatment with antinflammatory drugs or steroids. This incidence is higher than that recorded (7%) among nonpregnant patients during 1 year of follow up (Alarcón, 2000).

In a retrospective study on 81 patients with UCTD and 100 pregnancies (Zucchi et al., 2020), 13% flared during pregnancy or puerperium, 3% of which experienced more severe flares and developed SLE with renal impairment. In the remaining cases, cutaneous manifestations (3%), hematological abnormalities (3%), arthralgias (1%), venous thrombosis (1%), myositis (1%) and chest pain (1%), occurred. All women who become pregnant in a moment of disease activity flared during pregnancy or puerperium with the same clinical manifestations. Moreover, women with UCTD and anti-dsDNA antibodies showed an increased risk of flares and evolution to a specific CTD, especially SLE. Similar results were reported in case of high disease activity at conception. These findings highlight the importance to assess autoantibody profile in the pre- or peri-conception phase in order to estimate flare risk and program appropriate follow-up, and to plan pregnancy during disease remission.

**FIGURE 2:**
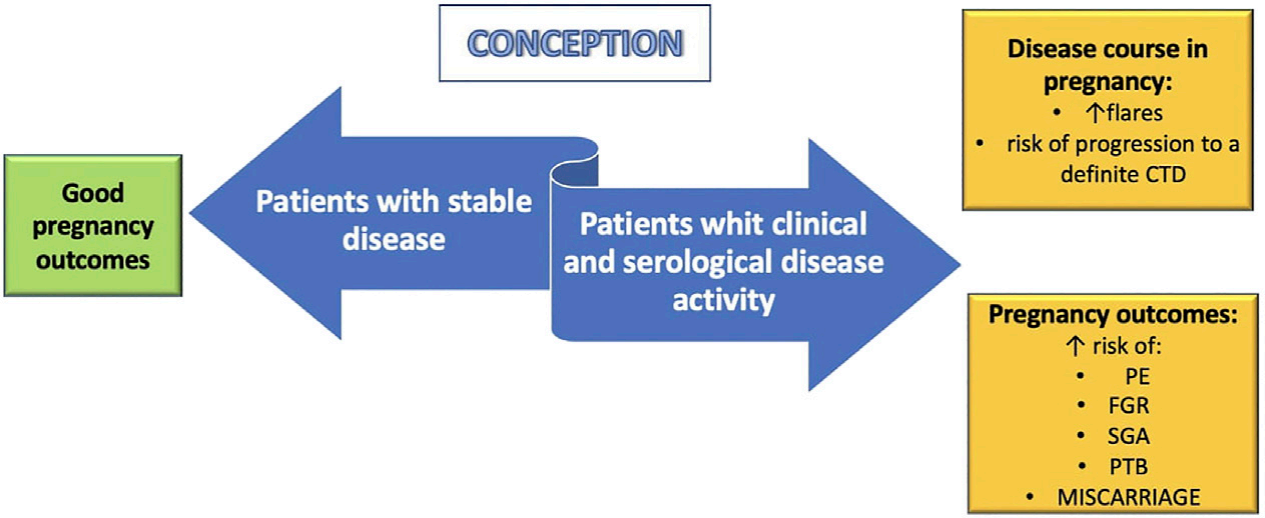
Pregnancy implications of undifferentiated connective tissue disease (UCTD). Women with stable UCTD at conception seem to have favorable maternal and fetal outcomes. On the contrary, patients with clinical and serological disease activity have an increased risk of flares, progression to a definitive connective tissue disease (CTD) and poor pregnancy outcomes. PE: preeclampia; FGR: fetal growth restriction; SGA: small for gestational age; PTB: preterm birth.

On the other hand, women with inactive or steady-state mild disease at conception have a low risk of flares during pregnancy (Figure 2) (Barnea et al., 2013). In case of SLE, current clinical guidance recommends conceiving at least after 6 months of disease quiescence (Götestam Skorpen et al., 2017). Although women with UCTD should be advised to conceive during inactivity of the disease to reduce the risk of relapse during pregnancy, the optimal length of the quiescent period before conception is not known.

## How UCTD Can Affect Pregnancy Outcomes?

UCTD is the most common rheumatic disorder diagnosed during pregnancy and may have a negative impact on pregnancy outcomes, as shown in Figure 2.

In a retrospective study by Zucchi et al. (2020), 11% of pregnancies ended in miscarriage in the first trimester and 29% experienced obstetric complications, including PTB (10%), small for gestational age (SGA) infants (10%), intrahepatic cholestasis of pregnancy (3%), preterm premature rupture of the membranes [pPROM] (2%), gestational diabetes [GDM] (2%) and PE (1%). In another case-control study (Spinillo et al., 2008b), the prevalence of pregnancy complications was 39% among women with UCTD versus 13.4% among healthy women. This prevalence was even higher in the subgroup of patients with UCTD and anti-Ro (SSA) (50%) antibodies, which are commonly detected in patients with UCTD (Alarcón, 2000; Mosca et al., 2006). Since fetuses exposed to maternal SSA and/or SSB antibodies can develop congenital heart block and neonatal lupus (Cavazzana et al., 2001; Motta et al., 2007), patients with UCTD should be tested for these antibodies before or during pregnancy (Spinillo et al., 2008b). The importance of studying the antibody profile of these women is also confirmed by the results of a multicentre retrospective cohort study conducted by Radin et al. (2020). Among 224 pregnancies, 177 (79%) ended in live births, 45 (20.1%) in miscarriages and 2 (0.9%) in stillbirths. Moreover, 2.2% of these women experienced PE, 4.9% gestational hypertension, 5.4% GDM, 16.8% PTB and 11.9% SGA. The authors also found that women with aPL and ENA antibodies had an increased risk of miscarriages and stillbirths (*p* < 0.05).

In a prospective cohort study by Beneventi et al., it was reported that bilateral uterine artery (UtA) notching at first, second and third trimester Doppler assessments was more frequent in women with UCTD compared to controls and was associated with adverse pregnancy outcomes. These results indicated that inadequate invasion of trophoblast is common among pregnant women with UCTD (Beneventi et al., 2012). It was speculated that antibodies may have a direct consequence of defective placentation and poor pregnancy outcomes (Spinillo et al., 2016). In particular, ANA may affect oocyte quality and embryo development, reducing the implantation rates (Ying et al., 2013), and may induce adverse pregnancy outcomes via the activation of the complement cascade (Veglia et al., 2015). Some experimental data (Habara et al., 2002; Ferreira et al., 2007; Pietropolli et al., 2015) have also suggested that ANA may compromise uterine blood flow in non-pregnant women who experienced recurrent pregnancy loss (RPL): an impaired uterine perfusion, in fact, may affect the endometrial receptivity and cause early pregnancy complications. Moreover, endothelial dysfunction (Laczik et al., 2014) and cytokine imbalance (Nakken et al., 2015) were found in pregnant women with UCTD, playing a role in causing defective placentation. Therefore, ANA and inflammation may affect platelet function, imbalance coagulation/anticoagulation mechanisms promoting a prothrombotic state, cause endothelial dysfunction and, ultimately, may impair spiral arteries remodeling and placenta development, increasing the risk of PE, FGR, SGA and RPL in pregnant women with UCTD (Yang et al., 2020).

## Disease Management and Prevention of Adverse Pregnancy Outcomes: What Are the Treatments of Choice in Pregnancy?

Nonsteroidal anti-inflammatory drugs (NSAIDs), corticosteroids and antimalarial drugs are the most common treatments with a satisfactory safety profile in pregnancy. In the most severe cases, especially in women with organ injuries and in those with poor response to other treatments, immunosuppressive drugs should be considered (Marwa and Anjum, 2021). Immunotherapy may also prevent placental insufficiency by regulating immune system and vascular function. Therefore, immunosuppressant do not only improve symptoms and prevent disease recurrence and progression, but may also decrease adverse pregnancy outcomes (Yang et al., 2020). However, the treatment efficacy in pregnant women with UCTD has been poorly studied.

Methotrexate (MTX), leflunomide, and mycophenolate mofetil (MMF) are contraindicated in pregnancy and should be discontinued before pregnancy, at least 6 weeks in the case of MMF to at least 3 months in the case of MTX (Marder et al., 2016).

### Nonsteroidal Anti-Inflammatory Drugs

Nonsteroidal anti-inflammatory drugs (NSAIDs) are not associated with congenital malformations and are not contraindicated in the first and second trimester. However, with the exception of low-dose aspirin, they should be avoided in the third trimester due to the risk of premature closure of the ductus arteriosus. There are insufficient data to recommend the use of selective COX II inhibitors in pregnancy and, therefore, they should be discontinued (Flint et al., 2016a; Marder et al., 2016).

Aspirin, an acetylated salicylate classified among the NSAIDs, has anti-inflammatory, analgesic, antipyretic, and antiplatelet effects. During pregnancy, it not only may improve clinical manifestations of rheumatic disease and other inflammatory disorders but may also be used for the management of antiphospholipid syndrome and for the prevention of PE. Salicylates in low doses are compatible with nursing, but high-dose aspirin should be avoided (Bermas, 2020).

### Corticosteroids

Corticosteroids can be safely prescribed in each trimester of pregnancy (level of evidence 1++, grade of recommendation A) (Flint et al., 2016a). While fluorinated glucocorticoids (e.g., dexamethasone and betamethasone) cross the placenta and can be used for prenatal lung maturation, nonfluorinated glucocorticoids (e.g., prednisone and prednisolone) cross the placental barrier just in small amounts. It was estimated that by administering 20 mg of nonfluorinated glucocorticoids, about 10% reaches fetal plasma. Therefore, prednisone and prednisolone at these doses are considered safe for treating symptomatic pregnant women with rheumatic disorders (Marder et al., 2016). Corticosteroids during pregnancy may increase the risk of premature rupture of the membranes (PROM), FGR and, in the mother, pregnancy-induced hypertension, GDM, osteoporosis, and infection (Østensen et al., 2006). Moreover, early evidence have suggested that glucocorticoid exposure during the first trimester increases the risk of fetal cleft palate (Park-Wyllie et al., 2000) by 3.4 times (Park-Wyllie et al., 2000). However, in a Danish cohort study evaluating corticosteroids exposure during the first trimester in 51,973 pregnancies, this association was not confirmed (Hviid and Mølgaard-Nielsen, 2011). Therefore, the lowest effective dose of glucocorticoids should be used to control disease activity during pregnancy, while high-dose schemes should be limited to women with organ-threatening disease (Bermas, 2020).

Corticosteroids in low doses are able to suppress T and natural killer (NK) cells and impair complement activation, which may allow adequate trophoblast invasion and placentation (Bansal, 2010; Tang et al., 2013). For these reasons, it was suggested that steroids at low dosage may improve pregnancy outcomes in women with CTDs (Bramham et al., 2011; Mekinian et al., 2017), especially in those with refractory obstetrical APS, although not all studies confirm this association (Sciascia et al., 2016). Data about the effect of corticosteroids on pregnancy outcomes in women with UCTD are even more limited. Only one study (Sun et al., 2021) explored the effect of prednisone among patients with unexplained RPL and positive ANA but without the required criteria to diagnose a definite CTD. The authors concluded that in these patients the combination of prednisone and aspirin was not more effective than aspirin alone as no significant difference between the two groups regarding live birth rates and pregnancy outcomes was reported. Further studies are needed to establish the effects of steroids on pregnancy outcomes in women with CTDs and UCTD.

Glucocorticoids are compatible with breastfeeding. They are excreted in breastmilk in low concentration, but it is advised discarding breast milk for the first 4 h following ingestion of a dose of prednisone ≥20 mg, as the peak concentration in breast milk is achieved 2 h after maternal ingestion (Bermas, 2020).

### Antimalarial Therapy

Hydroxychloroquine (HCQ) is the antimalarial drug most commonly used in symptomatic women with rheumatic disease at conception (de Jesús et al., 2020). Although HCQ crosses the placenta, most studies have confirmed fetal safety. Thus, it should be continued during pregnancy (Flint et al., 2016b). However, a recent cohort study comparing 2045 pregnancies exposed to HCQ with 3,198,589 controls showed an increased risk of major congenital malformations among women treated with HCQ during pregnancy (Huybrechts et al., 2021). Nevertheless, no particular pattern of malformations was identified and potential confounding factors, such as the use of other drugs, were not considered (Bermas and Chambers, 2021).

Several studies have also suggested that HCQ may improve disease course and pregnancy outcomes in women affected by CTDs (Buchanan et al., 1996; Costedoat-Chalumeau et al., 2003). In fact, HCQ seems to increase the live birth rate in pregnant women with persistent positive aPL (Tian et al., 2021), to reduce the incidence of FGR (Canti et al., 2021) and other obstetric complications such as PE, pregnancy hypertension and PTB (Duan et al., 2021) in those with SLE, and to decrease the recurrence rate of congenital heart block in anti-SSA/Ro-positive mothers (Izmirly et al., 2020). However, the effect of HCQ in pregnant patients with UCTD is poorly investigated. Preliminary data have suggested good pregnancy outcomes and high birth rates when UCTD patients with RPL were treated with a combination of HCQ, low-dose prednisone and anticoagulation. An ongoing three-arm, multicenter, open-label randomized controlled trial (NCT03671174) is studying pregnancy outcomes and disease course of UCTD patients with RPL using HCQ combined with low-dose prednisone, aspirin and Low Molecular Weight Heparin (LMWH) versus aspirin and LMWH alone or hydroxychloroquine combined with ASA and LMWH (Yang et al., 2020).

HCQ should also be used safely during breastfeeding (Bermas, 2020).

Available evidence about chloroquine is even more limited, but it appears to be safe in pregnancy (Marder et al., 2016).

### Steroid-Sparing Immunosuppressive Medications

If the symptoms are severe or persist despite the treatments described above, immunosuppressant agents should be considered (Marwa and Anjum, 2021). Azathioprine (AZA), sulfasalazine (SSZ), tacrolimus and cyclosporine (CSA) are compatible with pregnancy and breastfeeding (Marder et al., 2016; Marwa and Anjum, 2021). However, no study has specifically investigated the use of these drugs in pregnant women with UCTD.

AZA is one of the most studied and prescribed immunosuppressant drugs in pregnancy. It is a purine metabolism antagonist, that interferes with DNA synthesis. AZA is a prodrug and is converted post administration into a pharmacologically active metabolites, not all of which cross the placental barrier (Marder et al., 2016). Some studies have found an association between AZA and poor pregnancy outcomes, such as congenital malformations, PTB, FGR, neurocognitive deficits and transient immunological disfunctions. However, these findings mostly resulted from retrospective studies or case reports, with low quality of evidence. Moreover, whether these complications arise from AZA assumption or from the underlying disease or other concomitant treatments cannot be established (Belizna et al., 2020). More recent data did not confirm the association between AZA and pregnancy complications (Saavedra et al., 2015; Kanis et al., 2017). Further studies providing long-term follow-up and considering influencing factors such as maternal characteristics, disease activity and concomitant treatments are needed to clarify this field (Belizna et al., 2020).

SSZ possesses both antinflammatory properties mediated by its 5-aminosalicylic acid moiety and antibacterial characteristics (Bermas, 2020). SSZ and its metabolite sulfapyridine cross the placenta; however, no increase in miscarriage or congenital malformations are reported (Marder et al., 2016). SSZ inhibits absorption and metabolism of folic acid; therefore, some guidelines recommend taking folic acid supplement of 5 mg per day during pregnancy (Flint et al., 2016b; Marder et al., 2016). SSZ is transferred in low concentration in milk and is considered compatible with breastfeeding in healthy, full-term infants. However, women taking SSZ should avoid breastfeeding premature infants or those with hyperbilirubinemia or glucose-6-phosphate dehydrogenase (G6PD) deficiency (Bermas, 2020).

Tacrolimus is a calcineurin inhibitor. Although its use is compatible with pregnancy (Flint et al., 2016b), there are relatively few data concerning the effect of tacrolimus on pregnancy and its long-term immunomodulatory effects on the offspring (Bermas, 2020). Thus, pregnant women should be treated with the lowest effective dose of tacrolimus, the levels of which should be monitored regularly to avoid toxicity (Marder et al., 2016).

CSA is also a calcineurin inhibitor. There are conflicting reports on the transfer of CSA across the human placenta. Some reports have found little or no transfer, while others have found comparable CSA levels in the placenta and in maternal blood (Bermas, 2020). Since it does not appear to be associated with congenital defects and other pregnancy complications, it should be taken during gestation at the lowest effective dose, monitoring maternal blood pressure and renal function (Flint et al., 2016b; Marder et al., 2016).

### Biologic Agents

There are only few data regarding the safety of biologic agents in pregnancy (Bermas, 2020).

The majority of tumor necrosis factor (TNF) inhibitors (infliximab, etanercept, adalimumab, and golimumab) contain the Fc portion of IgG1 that crosses the placental barrier only in small amounts during the first and second trimester. Conversely, placental transfer during the third trimester increases and higher levels of these drugs can be detected in fetal circulation. Therefore, if possible, these treatments should be discontinued in the third trimester. On the contrary, the tumor necrosis factor inhibitor certolizumab does not contain an Fc region, its passage through the placenta is minimal and its use is compatible with each trimesters of pregnancy (Flint et al., 2016b; Sammaritano et al., 2020).

Limited data exist regarding the safety during pregnancy of rituximab, anakinra, ustekinumab, tocilizumab, abatacept and belimumab. Therefore, alternative medications should be considered during pregnancy (Marder et al., 2016).

Rituximab is a chimeric monoclonal antibody used as a therapeutic biologic agent in RA, as well as in other autoimmune and lymphoproliferative disorders. It leads to peripheral B-cell depletion through targeting the CD20 antigen present on B lymphocytes (Bermas, 2020). This medication seems to be responsible for hematologic abnormalities in neonates but not for congenital anomalies (Chakravarty et al., 2011). It has been demonstrated a complete but transient B cell depletion in the child of a mother treated with rituximab for Burkitt lymphoma (Friedrichs et al., 2006), but this association was not confirmed in others case reports of women who received rituximab in the first and second trimester of pregnancy (Herold et al., 2001; Kimby et al., 2004)*.* Although it is recommended discontinuing rituximab for 1 year prior to conception, whether rituximab is transferred through the placenta before 12 weeks of gestation is not known. Therefore, if indicated, this medication can be used at conception. Moreover, in life-threatening conditions, it can be used throughout pregnancy after a detailed discussion of the potential risks and benefits of such therapy. As transmission in breast milk is low, women receiving rituximab can breastfeed their infants (Bermas, 2020).

### Vitamin D

Vitamin D, a fat-soluble vitamin, regulates not only calcium absorption and bone metabolism, but also the immune system (Zold et al., 2011). In fact, it modulates differentiation and activation of CD4^+^ lymphocytes, increases the number and function of Treg cells, inhibits the differentiation of monocytes and dendritic cells (DCs), stimulates the function of Th2 cells and reduces proinflammatory cytokines, such as interferon (IFN)-γ, interleukin (IL)-2, and tumor necrosis factor (TNF)-α produced by Th1 cells (Cutolo and Otsa, 2008; Szodoray et al., 2008; Zold et al., 2011). Several studies have showed that vitamin D deficiency is involved in several autoimmune disorders (Merlino et al., 2004; Ruiz-Irastorza et al., 2008; Lee and Bae, 2016) and that its supplementation may reduce disease activity (Antico et al., 2012). It is well known that UCTD patients have immune abnormalities, such as high IFN *γ* levels, a decreased number of Tregs and an increased number of Th 17 cells. It was also shown that patients with UCTD had lower levels of 25(OH) D vitamin compared to healthy individuals (Zold et al., 2008) and that its supplementation can restore the Th17/Treg imbalance (Zold et al., 2011). Since a role of T cells in achieving and maintaining pregnancy has been suggested, a link between vitamin D levels and pregnancy outcomes in women with autoimmune disease, including UCTD, could exist.

Hypovitaminosis D was associated with poor pregnancy outcomes such as miscarriage, PE and FGR. Consequently, checking vitamin D levels before and during pregnancy should be considered in women at high risk of vitamin D deficiency, such as in those with autoimmune diseases, in order to implement appropriate treatment (Cyprian et al., 2019). Although available data suggest a role of hypovitaminosis D in the pathogenesis of adverse pregnancy outcomes, only small and non-controlled studies have investigated the potential role of vitamin D supplementation in pregnant women with autoimmune disease (Alijotas-Reig, 2013; García-Carrasco et al., 2018; Cohen et al., 2020). Therefore, randomized and controlled trials are necessary to establish whether vitamin D treatment could be useful in pregnant patients with UCTD and whether it may prevent the development to a defined CTDs and/or may improve pregnancy outcomes (Zold et al., 2011).

## Should Aspirin and Low Molecular Weight Heparin Play a Role to Improve Pregnancy Outcomes?

Low-dose aspirin is recommended for pregnant women with SLE or APS to prevent pregnancy-induced hypertension (Author anonymous, 2018). However, it may also be considered in pregnant women with other autoimmune diseases and concomitant patient-specific risk factors. The mere presence of aPL, regardless of previous thrombotic events and/or previous negative obstetric outcomes, seems to be associated with a higher risk of PE. Therefore, prophylactic treatment with aspirin should be taken into account in pregnant women with UCTD and positive aPL, even if they do not fulfil criteria for APS (Sammaritano et al., 2020). Several studies have showed an improvement of pregnancy outcomes using prednisone and aspirin in ANA positive women (Zhu et al., 2013; Fan et al., 2016; Sun et al., 2021). However, this finding should be verified in further prospective randomized studies.

LMWH have been traditionally administered for its anticoagulant activity. However, it also has anti-inflammatory and immunomodulant properties, which may support embryo implantation and placentation (Zullino, 2021). Current guidelines recommend antepartum LMWH and aspirin prophylaxis for pregnant women with APS—whether obstetric or thrombotic—to improve pregnancy outcomes and/or reduce risk of thrombosis (Sammaritano et al., 2020).

Bruno et al. (2020) found that LMWH may improve vascularization index values of the uterine arteries in ANA-patients with a history of RPL. These findings suggest that LMWH might improve placentation and therefore pregnancy outcomes in women with UCTD. However, there is insufficient evidence to suggest the standard use of LMWH for women with autoimmune disease (Herold et al., 2001), including UCTD, and further studies are needed to confirm these results.

## Counseling During Conception, Pregnancy, and Breastfeeding

Pregnancy in women with UCTD carries a higher maternal and fetal risk compared with healthy women (Spinillo et al., 2008b; Castellino et al., 2011). Therefore, these women should be referred to a high-risk pregnancy center for preconception counseling and pregnancy planning whenever possible, and once pregnant for subsequent obstetric care.

Maternal and fetal outcomes are better when the disease has been quiescent prior to the pregnancy (Barnea et al., 2013; Zucchi et al., 2020). Therefore, a good disease control before conception is crucial to reduce pregnancy complications.

The preconception evaluation should include an assessment of disease activity and major organ involvement. Women with UCTD should be counseled carefully about their individual risk profile, with clear discussion of the morbidity and mortality risks to both mother and fetus during pregnancy. Medical treatments must be reviewed and adjusted prior to conception with the goal of maintaining medications that are compatible with pregnancy and discontinuing those that are contraindicated (Bermas, 2020). Anti-Ro/SSA and anti-La/SSB antibodies, aPL and anti-dsDNA antibodies should be assessed prior to pregnancy, as they were associated with pregnancy complications (Radin et al., 2020; Zucchi et al., 2020). Antithyroperoxidase (anti-TPO) and antithyroglobulin (anti-TG) antibodies should also be tested as they are rather common among UCTD subjects and have been associated with increased risk of RPL, congenital hearth block, and other complications of pregnancy (Beneventi et al., 2015; Spinillo et al., 2017).

Moreover, previous poor obstetric outcomes, such as FGR, PE, stillbirth, miscarriage, and PTB, should be taken into account in order to intensify follow-up during pregnancy and consider prophylactic treatments, like low-dose aspirin or LMWH (Author anonymous, 2018; Sammaritano et al., 2020).

Management of pregnant women with UCTD should require an individualized and multidisciplinary approach including obstetricians and rheumatologists. Early and stable UCTDs represent significant risk factors for poor reproductive outcomes, and similar to other rheumatic diseases, increased maternal and fetal surveillance is needed (Spinillo et al., 2017). The optimal monitoring schedule during pregnancy is not yet known. However, women with risk factors or poor prognostic indicators may require more frequent monitoring. It was suggested that a rheumatologic evaluation including clinical assessment and laboratory tests should be carried out every 4–8 weeks, while obstetric clinical evaluations should be performed monthly. The frequency of ultrasound scans with fetal biometry and Doppler assessment should be scheduled according to the autoantibody positivity, severity of disease and Doppler pulsatility index of uterine arteries (Spinillo et al., 2017). In anti-Ro/SSA-positive patients, fetal echocardiography should be performed (Friedman et al., 2008). As pregnancy and puerperium may favor disease relapse and evolution to a definitive CTD (Castellino et al., 2011; Yang et al., 2020), women with UCTD are recommended to inform clinicians about the onset of new signs and symptoms.

Medical treatments should be also reviewed in puerperium in order to evaluate the safety of medications in lactation (Bermas, 2020).

## Conclusion

Although UCTD is the most common rheumatic disorder diagnosed in pregnant women, data on the disease course, on maternal-fetal outcomes and on treatment during pregnancy are very limited.

Pregnancy represents a possible trigger for disease relapses and development of a defined CTD. Moreover, an increased risk of PE, FGR, PTB and other adverse pregnancy outcomes was reported in these patients. Women with disease activity at conception and with multiple antibodies positivity should be closely followed-up during pregnancy because of the risk of severe disease course and adverse pregnancy outcomes. Planning pregnancy, in addition to careful serological assessment and strict rheumatological and obstetric follow up, are mandatory to improve maternal and neonatal outcomes. NSAIDs, corticosteroids, antimalarial and immunosuppressive drugs may be used to reduce disease activity and pregnancy complications.

Vitamin D supplement in pregnant women with hypovitaminosis D and low dose aspirin in those with concomitant autoimmune disease and/or other risk factors should be considered in order to prevent unfavorable obstetric outcomes.
